# Personal

**Published:** 1916-12

**Authors:** 


### PERSONAL

Dr. R. P. Bush of Horseheads has been re-elected to the Assembly from Chemung County. Dr. Bush has always been foremost in promoting laws to benefit the health of the people.

Dr. A. W. Booth of Elmira has been elected president of the State Board of Medical Examiners.

Dr. Homer J. Trotter (Buffalo 1915) has completed his interne service in N. Y. City and has opened an office at 473 Virginia Street, Buffalo. His practice will be limited to diseases of the ear, nose and throat.

Dr. R. M. Sterrett, many years associated with the advertising of Antiphlogistine, sends greeting to his many friends, announcing his resignation as Advertising Manager of the Denver Chemical Mfg. Co., effective January 1st.

Dr. Julius Richter of Buffalo announces the removal of his offices to 2556 Main Street. Hours 9 and 1-2.

---

Drs. George W. Goler, Wesley T. Mulligan and John Aikman of Rochester attended the meeting of the New England Paediatric Society in Boston, Nov. 4.

---

Dr. and Mrs. Chas. W. Bethune of the Mathewson Apartments, 294 S. Elmwood Avenue, Buffalo, made a two-weeks trip in November to Washington, Baltimore, Boston, Philadelphia and New York, Dr. Bethune attending various clinics in these cities.

---

Dr. Jesse N. Roe of Buffalo announces the removal of his office to 481 Franklin Street, his practice being limited to dermatology and contagious diseases.

---

Dr. W. Gilman Thompson, Prof. of Medicine at Cornell University Medical College in N. Y. City, since the organization in 1898, has resigned, being succeeded by Dr. Lewis Atterbury Connor.

---

Dr. L. O. Thoma has moved from Buffalo to Forks, N. Y.

---

Dr. A. F. Luhr of Buffalo has moved his office to 519 Franklin Street.

---

Dr. S. Y. Howell of Buffalo spent the last two weeks of October hunting in the Adirondacks.

---

Dr. Walter L. Savage of Buffalo has moved to 1420 Amherst Street.

---

Dr. Francis E. Fronczak of Buffalo has been elected a director of the Am. Assn. for the Study and Prevention of Infant Mortality, which met in Milwaukee in October.

---

Dr. W. B. Whitcombe of Batavia having waived reappointment as town Health Officer, Dr. Henry M. Spofford has been appointed to this position.

---

The Board of Trustees of the Buffalo Orphan Asylum have elected Dr. Milton Bork to act as physician to the asylum.

---

Dr. Elmer E. Williams, University of N. Y. 1889, who has been in practice in N. Y. City, has recently moved to Syracuse, 814 Lancaster Avenue.

